# Editorial: Dynamic Functioning of Resting State Networks in Physiological and Pathological Conditions

**DOI:** 10.3389/fnins.2020.624401

**Published:** 2020-12-16

**Authors:** Filippo Cieri, Nicoletta Cera, Alessandra Griffa, Dante Mantini, Roberto Esposito

**Affiliations:** ^1^Department of Neurology, Cleveland Clinic Lou Ruvo Center for Brain Health, Las Vegas, NV, United States; ^2^Center for Psychology at University of Porto (CPUP), Faculty of Psychology and Educational Sciences, University of Porto, Porto, Portugal; ^3^Department of Clinical Neurosciences, Division of Neurology, Geneva University Hospitals and Faculty of Medicine, University of Geneva, Geneva, Switzerland; ^4^Institute of Bioengineering, Center of Neuroprosthetics, Ecole Polytechnique Fédérale De Lausanne (EPFL), Geneva, Switzerland; ^5^Research Center for Motor Control and Neuroplasticity, KU Leuven, Leuven, Belgium; ^6^Brain Imaging and Neural Dynamics Research Group, Istituto di Ricovero e Cura a Carattere Scientifico (IRCCS) San Camillo Hospital, Venice, Italy; ^7^Titano Diagnostic Clinic, Falciano, San Marino; ^8^Area Vasta 1, ASUR Marche, Pesaro, Italy

**Keywords:** default mode network, advanced neuroimaging, anticorrelations, dynamic brain activity, resting state networks (RSNs)

## Introduction

Modern neuroimaging techniques, such as Magnetic Resonance Imaging (MRI), allow for study of the brain from structural and functional perspectives. Particularly, resting state networks' (RSNs) connectivity explores the integration of activity across distant brain areas. In recent years a growing number of studies have shown that the resting state may provide a sensitive and valuable tool able to study cerebral functioning in normal and pathological conditions.

Cerebral activity recorded during cognitive tasks shows a baseline low frequency fluctuation at 0.01–0.1 Hz (Cordes et al., 2001). During rest, this baseline fluctuation organizes in a network of coordinated cerebral activity named the Default Network (DN), spanning the medial prefrontal, posterior cingulate, inferior parietal, and hippocampal cortices (Raichle et al., 2001; Andrews-Hanna et al., 2014). In recent decades, this network has received great attention because it contains several regions that support cognitive functions and undergo critical changes upon aging, cognitive decline, and neuropsychiatric disorders. The DN has been associated with reflective activity and self-referential mental processes and has been extensively characterized in neurological, psychiatric, and psychotherapeutic contexts (Cieri and Esposito, 2018, 2019). To date, in addition to the DN, at least 10 resting state networks have been identified. Among these, the Dorsal Attention Network (DAN) comprises regions commonly activated in attention demanding tasks. The DN and DAN show a pattern of anticorrelated activity in both task and resting state studies, suggesting that they are intrinsically organized into anticorrelated networks (Esposito et al., 2017).

The overall goal of this Research Topic was to provide a comprehensive coverage of the latest advances in dynamic functioning of RSNs both in physiological and neuropsychiatric conditions. The studies have used different neuroimaging techniques to probe functional connectivity (FC) in the brain, including resting-state functional MRI (rs-fMRI), Electroencephalography (EEG), Magnetoencephalography (MEG), and Positron Emission Tomography (PET).

## Neurodegenerative Diseases

Nine articles submitted to this Research Topic investigate FC changes occurring in physiological and pathological aging, with particular attention given to functional plasticity mechanisms and functional markers of prodromal dementia syndromes or conversion to dementia. The articles also highlight how the integration of multimodal functional imaging data (e.g., simultaneous EEG-fMRI recordings) and the analyses of dynamic functional connectivity (dFC) features, as well as subject-level molecular connectivity networks, may contribute to broadening our understanding of neurodegenerative disorders.

### Aging, Mild Cognitive Impairment, and Alzheimer's Disease

Alzheimer's Disease (AD) is the most common form of dementia, and is becoming increasingly common in our increasingly older societies. AD is characterized by several brain changes including β-amyloid and tau proteins accumulation, synaptic dysfunctions, and brain atrophy, especially in the medial temporal lobe. In addition to these modifications, a loss of cognitive functions (mostly in the memory domain) and of independence in daily activities are observed. The transition from healthy aging to AD is usually not immediate and passes through different phases, such as Significant Memory Concerns (SMC) and Mild Cognitive Impairment (MCI). Functional imaging may play a crucial role in identifying early mechanisms predictive of dementia onset.

Hojjati et al. integrate rs-fMRI FC and structural MRI features to predict the conversion of MCI patients to AD. By investigating MCI converter patients, MCI non-converter patients, AD patients, and healthy controls (HCs), the authors show the power of integrating multi-modal MRI data for the identification of early-stage AD.

Cera et al. compare FC patterns of the cingulate cortex between a sample of MCI patients and HCs with comparable levels of education. The authors explore RSNs activities, mapping the FC patterns of different subregions of the cingulate cortex. The cognitive decline observed in MCI patients relates to the global FC of the cingulate cortex, suggesting that the analysis of the cingulate cortex FC could be a helpful tool to better understand the brain mechanisms underlying MCI.

Dimitriadis et al. introduce a novel approach to identify MCI through MEG resting-state data, estimating a dFC graph using the imaginary part of phase lag value for both intra-frequency and cross-frequency couplings. This work shows how the adaptation of neuroinformatic tools combining advanced signal processing and network neuroscience elements can properly highlight the non-stationarity of time-resolved FC patterns, revealing a robust biomarker for MCI.

Bubbico et al. explore cerebral plasticity induced by learning a new language in elders, investigating how cognition together with functional brain organization can be improved late in life. This study analyzes the functional effects of a 4-month second language learning program in a group of HCs. After the program, in the intervention group, there are significant improvements in global cognition together with increased FC in the right frontal gyrus and left superior parietal lobule.

Caldwell et al. investigate how gender moderates typical biomarkers of AD. The authors employed group independent component analysis (ICA) to analyze rs-fMRI data from the Alzheimer's Disease Neuroimaging Initiative dataset. Results suggest that stronger anterior/posterior DN connectivity may support verbal learning in women at risk for AD dementia.

Feng et al. investigate the correlation between hippocampal FC and MRI radiomic features in AD. The AD group showed abnormalities of FC levels in the bilateral hippocampal functional network relating to hippocampal radiomic features.

### Parkinson's Disease

Evangelisti et al. explore the effects of L-dopa administration in early-stage Parkinson's Disease (PD) patients on FC patterns revealed by simultaneous recording of fMRI and EEG data. This pilot study provides a first insight into the potentiality of simultaneous EEG-fMRI acquisitions in PD patients, showing for both techniques the analogous direction of increased FC after L-dopa intake, mainly involving motor, dorsal attention, and the DN.

### Neuroimaging of Neurodegenerative Diseases: Positron Emission Tomography and Magnetic Resonance Imaging

Two interesting reviews were submitted about neurogenerative diseases providing an exhaustive overview of literature on rs-fMRI and PET imaging.

Sala and Perani summarize available evidence in the field of PET molecular connectivity, offering an overview of how this approach may broaden our understanding of the pathogenesis of neurodegenerative diseases, over and above “traditional” structural/functional connectivity studies. The review gives focus to the available strategies to investigate molecular connectivity at the single-subject level, of potential relevance for both research and diagnostic purposes.

Filippi et al. summarize the main currently available approaches for dFC analysis and report the recent application of these methods for the assessment of the most common neurodegenerative conditions, including AD, PD, dementia with Lewy bodies, and frontotemporal dementia. The authors point out the key role of dFC analyses, highlighting both technical and clinical aspects.

## Emotional Disturbances and Psychiatric Diseases

One of the targets of neuroscience, and particularly of advanced neuroimaging techniques, is to identify specific biomarkers associated with neurological disorders. These biomarkers have induced a great progression in modern cognitive neuroscience but remain elusive in psychiatric disorders due to their clinical heterogeneity and comorbidities. A collection of thirteen studies of this Research Topic highlights the different functional changes occurring in a variety of psychiatric, emotional, learning, eating, and sleep disorders, possibly contributing to the identification of disease-specific biomarkers. Moreover, some of these studies show how new neuroimaging measures quantifying functional connectivity dynamics, rest-task transitions, or network information flow may provide a finer-grain characterization of brain functioning in psychiatric conditions. Finally, particular attention is given to the identification of brain functional features reflecting specific behavioral/cognitive/emotional traits (e.g., reading abilities) or clinical symptoms (e.g., verbal auditory hallucinations or depressive symptoms), tackling clinical heterogeneity and inter-individual variations in psychiatric conditions.

### Depression

Damborská et al. explore whether resting-state EEG microstate temporal features can capture large-scale brain network dynamics relevant to depressive symptoms in patients with moderate to severe depression in bipolar affective disorder, depressive episode, and recurrent depressive disorder compared to HCs. Results suggest that the interindividual differences in resting-state microstate parameters could reflect altered large-scale brain dynamics relevant to depressive symptomatology during depressive episodes.

Wolff et al. conduct a combined rest and task EEG study in acute depressed major depression disorder (MDD) patients, compared to HCs. Results show that resting-state dynamics are atypical in MDD and strongly shape subsequent stimulus-induced activity. The authors conclude that MDD is characterized by alterations of the rest-stimulus interaction.

### Schizophrenia

Salman et al. employ a new information theoretic framework named dynamic functional domain connectivity (DFDC) to analyze resting-state dFC and the amount of information shared among functional domains in schizophrenia patients (SZ) and HCs. Functional domains are defined as subsets of functional networks, and their properties and interactions are quantified with entropy and mutual information measures. The authors show that the DFDC pairs tend to function in a more independent manner in SZ patients compared to HCs, suggesting higher uncertainty and randomness in SZ brain function.

Liu et al. explore hippocampal dysconnectivity in SZ patients and its correlation with auditory verbal hallucinations. This study suggests that locations in the hippocampus mediate the neural mechanism behind auditory verbal hallucinations in SZ.

Li et al. review functional network changes occurring in SZ and epilepsy to highlight possible similarities and differences. In light of the reviewed literature, the authors question whether electroconvulsive therapy (ECT), one of the oldest therapeutic modalities in psychiatric clinical practice, relies on antagonistic and/or affinitive mechanisms between these two disorders. The authors highlight how the study of RSNs, such as the default, salience, and dorsal attention networks, has provided a new perspective to understand the relationship between schizophrenia and epilepsy and has shown how ECT modifies the dynamics of these brain networks.

### Borderline Personality Disorder

Emerging evidence supports the hypothesis that emotional dysregulation results from aberrant connectivity within the fronto-limbic neural networks in patients with borderline personality disorder (BPD). Despite its important role in emotional regulation, the anterior cingulate cortex (ACC) has not yet been fully explored in BPD patients. Using seed-based resting-state FC and probabilistic fiber tracking, Lei et al. explore the alterations of functional and structural connectivity of the ACC in young non-medicated BPD patients compared to HCs. The authors show that ACC structural and functional connectivity alteration underlie the deficient emotional regulation circuitry of BPD patients. Such alterations may be important biomarkers of BPD and point to potential BPD treatment targets.

### Phobia, Somatization Disorder, Psychogenic Erectile Dysfunction

Indovina et al. test the hypotheses that individuals with agoraphobic symptoms have visual-vestibular network alterations similar to those of patients with persistent postural perceptual dizziness, and that these alterations are influenced by neuroticism and introversion. They find that the FC of two brain networks is lower in subjects with subclinical agoraphobia as compared to HCs. These networks integrate visual vestibular and emotional responses to guide movement in space.

Ou et al. investigate the seed-based nucleus accumbens (NAc) FC in first-episode, drug-naive patients with somatization disorder. This study reveals that patients have increased NAc connectivity within the frontal regions of the reward circuit. Increased left NAc-right gyrus rectus connectivity can be used as a potential marker to discriminate patients with somatization disorder from HCs.

Previous studies have illustrated neural changes in patients with psychogenic erectile dysfunction, while only a few works have focused on the neural underpinning of the psychosocial status in patients with this dysfunction. Yin et al. investigate associations among altered cerebral activity patterns, impaired erectile function, and disrupted psychosocial status in patients and HCs, pointing out the key role of psychosocial disorders with respect to the neural changes observed in psychogenic erectile dysfunction.

### Eating Disorders: Anorexia Nervosa

Anorexia nervosa (AN) is a severe psychopathology characterized by intense fear of gaining weight, relentless pursuit of thinness, deep concerns about food, and a pervasive disturbance of body image. Nowadays, eating disorders are more widespread and characterized by diagnostic fluidity with other eating disorders, carry a high psychiatric comorbidity burden, and are associated with elevated suicide risk (Welch et al., 2016). FMRI studies try to shed light on the neurobiological underpinnings of these disorders (Esposito et al., 2018).

Collantoni et al. use a graph-theory approach to explore FC differences between AN patients and HCs, focusing on the effect of serotonin transporter (5-HTTLPR) genotype on regional and global network characteristics. AN patients display lower network clustering and altered hub distribution compared to HCs. Moreover, carriers of the short allele are characterized by lower small-world and modularity indexes in the patient group, while an opposite trend is present in HCs.

### Sleep Disorders: Primary Insomnia

Wu et al. compare the network properties of the structural and functional connectomes derived from diffusion MRI and rs-fMRI data of primary insomnia patients and HCs. They investigate the relationship between abnormal network metrics and clinical characteristics, including disease duration, sleep quality, and anxiety and depression indexes. Patients show small-world architecture with lower global and local efficiencies compared to HCs and present five disrupted subnetworks in the limbic cortico-basal-ganglia circuit and left DN. These results suggest that abnormalities of brain network architecture may be closely linked to the clinical characteristics of primary insomnia.

### Post-traumatic Stress Disorder

Major adverse events trigger different kinds of emotional dysfunctions or psychiatric disorders in the exposed subjects. Recent literature shows that exposure to natural disasters such as earthquakes can generate difficulties in identifying and describing feelings (alexitimia), correlated to the intensity of post-traumatic symptoms (Di Giacinto et al., 2015). Neuroimaging data show that trauma exposure is related to derangement of resting-state FC. Pistoia et al. investigate the neurofunctional changes related to the recognition of emotional faces in L'Aquila earthquake witnesses. The results show that, in earthquake-exposed subjects, there is a significant reduction in the correlation between the accuracy in recognizing facial expressions and the FC of the visual network and DN.

### Learning Disorder: Dyslexia

Nachshon et al. compare functional and cognitive features of children with reading difficulties and age-matched typical readers. A negative correlation between reading, emotional, and executive abilities is found in both groups. Children with reading difficulties show significantly decreased emotional and executive abilities, altered network efficiency within the emotional network, and lower FC between the amygdala and frontal pole regions. Stronger FC between the amygdala, pre-central, and post-central gyri related to worse reading, emotional, and executive abilities in both typical readers and children with reading difficulties.

## Other Clinical Conditions

### Multiple Sclerosis

In their review, Valsasina et al. describe the methods currently used to assess dFC from rs-fMRI data and summarize the main dFC findings in multiple sclerosis. An overview of the main results obtained in neurodegenerative and psychiatric conditions is also provided. The analysis of dynamic (or time-varying) FC contributes to providing significant information on intrinsic brain functional organization, both in healthy and diseased conditions, which complements data produced by static FC approaches. Time-varying FC seems to be an intrinsic property of the brain with a neural origin, although some open questions still remain about its correct interpretation.

### Chronic Inflammatory Bowel Disease: Crohn's Disease

Kornelsen et al. investigate differences in brain structure and function in patients with Crohn's disease compared to HCs. Voxel-based morphometry analysis is performed to contrast Crohn's disease and HCs' structural images. ROI analyses are run to assess FC for RSN nodes. ICA identifies whole brain differences in FC associated with RSNs. In patients, changes of FC associated with sex are observed in both ROI and ICA analyses and suggest an influence of Crohn's disease on brain function.

### Eye Diseases: Glaucoma

Minosse et al. explore a putative reorganization of functional brain networks in Glaucomatous patients and evaluate the potential of functional network measures as biomarkers of disease severity in terms of their relationship to clinical variables and select retinal layer thicknesses. The authors compare resting-state FC of glaucoma patients and HCs using disruption indices that measure the degree of overall reorganization of specific properties in the whole brain network. In Glaucoma, group-wise disruption indices are negative for all graph theoretical metrics. The disruption index of the clustering coefficient yielded the best discriminative power for differentiating patients from HCs. These results support a possible relationship between FC and disease severity in Glaucoma.

### Stroke

Kalinosky et al. describe brain connectivity associated with multisensory integration during wrist control in stroke survivors, age-matched HCs, and healthy young adults. They use a novel fMRI task paradigm involving wrist movement developed to gain insight into the effects of multimodal (visual and auditory) sensory feedback on brain function in stroke participants. Results show that stroke participants have greater contralesional activation than HCs during the visual feedback condition and less ipsilesional activity than HCs during the auditory feedback condition. Connectivity analyses between the lesioned sensorimotor cortex and the contralesional cerebellum demonstrate decreased FC in stroke participants, positively correlated to manual dexterity. These results suggest that task-based FC provides details on brain network reorganization in stroke survivors.

### Neonatal Pathology: Preterm Neonates

Tortora et al. evaluate FC changes in preterm neonates that underwent invasive procedures during the postnatal period and correlate them with the neurodevelopmental outcome at 24 months. The authors investigate two groups of preterm neonates: subjected and non-subjected to painful invasive procedures during neonatal intensive care. The results show that early exposure to pain is associated with abnormal FC of developing networks involved in the modulation of noxious stimuli in preterm neonates, contributing to the neurodevelopmental consequence of preterm birth.

### Neuropathic Pain: Post-herpetic Neuralgia

Huang et al. compare patients with herpes zoster, individuals with post-herpetic neuralgia, and HCs, using fMRI to explore the effects of these diseases on brain activity and to detect the neural mechanisms of cognitive impairment in neuropathic pain patients. The results show that spontaneous brain activity is reduced in both patient groups compared to HCs. In particular, patients have decreased ALFF in the precuneus, posterior cingulate cortex, and middle temporal gyrus. The authors conclude that ALFF values in pain-related regions can be used as an fMRI-based biomarker for the classification of subjects with different pain conditions.

## Brain Phisiology and Methodological Approaches

Five articles of this Research Topics tackle more general aspects of FC assessment, such as the influence on FC measures of data processing and analysis steps (e.g., spatial leakage correction and sliding window approaches), of the uncertainty of oxygen consumption quantification, and of additional factors specific to the investigated species (human or non-human primates).

Della Penna et al. evaluate the impact of the geometric correction scheme (GCS) on MEG functional topology at rest. Source-projected MEG signals are affected by spatial leakage, leading to the estimation of spurious, blurred connections that may affect the topological properties of brain networks. To reduce leakage effects, several correction schemes have been proposed, including the GCS. The authors explore the impact of GCS correction on classical graph measures used to describe the architecture brain functional networks by comparing such measures between GCS-corrected and uncorrected MEG connectomes. The use of GCS considerably reorganizes the topology of connectivity, reducing within-hemisphere interactions mainly in the beta and gamma bands and increasing cross-hemisphere interactions mainly in the alpha and beta bands. Overall, the GCS leakage correction removes spurious local connections, but confirms the role of dynamic hub regions (specifically, the anterior and posterior cingulate cortices) in integrating information in the brain at rest.

Liuzzi et al. propose another interesting MEG study on FC, using multivariate autoregressive and neural mass models with *a priori* defined ground truths to systematically analyze the sensitivity of conventional metrics in combination with different window lengths to detect genuine fluctuations in connectivity for various underlying state durations. The authors show that fixed sliding window connectivity approaches can detect modulations of connectivity, but mostly if the underlying dynamics operate on moderate to slow timescales. In practice, this can be a drawback, as state durations can vary significantly in empirical data.

In their mini review Watabe and Hatazawa discuss the value of PET imaging to evaluate FC. Previous studies have assessed RSNs mainly based on spontaneous fluctuations in blood-oxygen-level-dependent (BOLD) fMRI signals. However, separation between regional increases in cerebral blood flow and oxygen consumption is theoretically difficult using BOLD-fMRI. Such a separation can be achieved using quantitative 15O-gas and water PET. In addition, 18F-FDG PET can be used to investigate FC based on changes in glucose metabolism, which reflects local brain activity. Previous studies have highlighted the feasibility and clinical usefulness of 18F-FDG-PET for the analysis of RSNs, and recent studies have utilized simultaneous PET/fMRI recordings for such analyses. PET and fMRI provide different types of information and integrating these modalities may help elucidate the pathological mechanisms underlying certain brain diseases and in characterizing individual patients.

Jovellar and Doudet discuss anatomical and functional differences between human brains and non-human primate brains that can affect pre-processing and analysis of fMRI data, anesthetic effects on BOLD signal and FC, and factors that can affect dynamic causal modeling application in fMRI. There are established preprocessing methods that prepare human fMRI data for subsequent analyses, such as dynamic causal modeling to infer effective connectivity; however, these are not optimized for non-human primate fMRI image analysis. The majority of fMRI imaging in non-human primates is done under anesthesia, which can decrease BOLD signal-to-noise ratio and spontaneous fluctuations. While dynamic causal modeling is a tool that can reliably ascertain directed causal influence (effective connectivity) and address enduring questions on fMRI hemodynamic responses, many uncertainties remain.

Complementarily, van Den Brink et al. review recent works investigating how neuromodulatory systems shape correlations of large-scale cortical activity fluctuations. They discuss functional studies in the human, monkey, and rodent brain and provide a structured but selective overview of these works, distilling a number of emerging principles. The authors underline that efforts to chart the effect of specific neuromodulators and, in particular, of specific receptors, on intrinsic correlations may help in identifying shared or antagonistic principles between different neuromodulatory systems. Such principles can inform models of healthy brain function and may provide an important reference for understanding cortical dynamic alterations observed in neurological and psychiatric disorders, potentially paving the way for mechanistically inspired biomarkers and individualized treatments.

Two studies explore new methodological approaches to characterize brain network centrality dynamics and dynamic effective connectivity.

Wink explores the use of centrality dynamics extracted from eigenvector centrality mapping of fMRI data for measuring group-differences in imaging studies. The analyses on the OpenNeuro dataset show that centrality dynamics can be used to identify age and gender between-group differences, and that age and gender distributions need to be considered in functional imaging studies.

Deshpande and Jia employ a dynamic multivariate autoregressive model to estimate dynamic effective connectivity (DEC), a method first validated on simulated data and then applied to real rs-fMRI data. The authors perform a dynamic clustering (adaptive evolutionary clustering) of DEC matrices across multiple levels -spatial locations, time, and subjects- which highlights a small number of directional brain network configurations akin to brain microstates, alternating over time in a quasi-stable manner. The dominant DEC networks involved spatially distributed brain regions mainly pertaining to memory, emotion, and executive and language functions. Finally, the authors use a larger cohort of rs-fMRI and behavioral data from the Human Connectome Project to show that metrics derived from DEC analysis can explain larger variance in 70 behavioral scores compared to static effective connectivity measures.

One study illustrates how FC analyses can be used to assess in real time the impact of external interventions on brain function. Tang et al. conducted a multi-session analysis combing transcranial magnetic stimulation (TMS) and fMRI experiments to explore the spatiotemporal effects of TMS within the fronto-hippocampal network. Ten healthy volunteers are modulated by intermittent theta-burst stimulation at a precise site within the left dorsolateral prefrontal cortex, navigated by individual structural MRI images. The findings suggest that the intermittent theta-burst stimulation effect dynamically changed over time, from local neural activations at the stimulated site to its connected remote regions within the fronto-hippocampal network.

Finally, in his hypothesis and theory contribution Northoff reflects on the relationship between neuronal activity, world-brain interactions, and mental features. Northoff proposes a change in our methodological strategy to approach the brain, from a pre-Copernican vantage point from within brain to a post-Copernican vantage point from beyond brain. This change would allow neuroscience to take into view what happens beyond the brain itself, e.g., the world, and how that shapes the brain and its neural activity, e.g., world-brain relation. This view converges with the free energy principle proposed by Karl Friston.

## Author Contributions

RE and FC conceived the topic. RE, FC, DM, AG, and NC managed and checked the review processes. RE and FC drafted the editorial and worked on the revisions with DM, AG, and NC. All authors contributed to the article and approved the submitted version.

## Conflict of Interest

The authors declare that the research was conducted in the absence of any commercial or financial relationships that could be construed as a potential conflict of interest.

## References

[B1] Andrews-Hanna J. R. Smallwood J. Spreng R. N. (2014). The default network and self-generated thought: component processes, dynamic control, and clinical relevance. Ann. N. Y. Acad. Sci. 1316, 29–52. 10.1111/nyas.12360 24502540 PMC4039623

[B2] Cieri F. Esposito R. (2018). Neuroaging through the lens of the resting state networks. Biomed. Res. Int. 2018:5080981. 10.1155/2018/5080981 29568755 PMC5820564

[B3] Cieri F. Esposito R. (2019). Psychoanalysis and neuroscience: the bridge between mind and brain. Front. Psychol. 10:1983. 10.3389/fpsyg.2019.01983 31555159 PMC6724748

[B4] Cordes D. Haughton V. M. Arfanakis K. Carew J. D. Turski P. A. Moritz C. H.. (2001). Frequencies contributing to functional connectivity in the cerebral cortex in “resting-state” data. AJNR Am. J. Neuroradiol. 22, 1326–1333. 11498421 PMC7975218

[B5] Di Giacinto A. Lai C. Cieri F. Cinosi E. Massaro G. Angelini V.. (2015). Difficulty describing feelings and post-traumatic symptoms after a collective trauma in survivors of L'Aquila earthquake. J. Ment. Health. 24, 150–154. 10.3109/09638237.2015.1019055 25989491

[B6] Esposito R. Cieri F. Chiacchiaretta P. Cera N. Lauriola M. Di Giannantonio M.. (2017). Modifications in resting state functional anticorrelation between default mode network and dorsal attention network: comparison among young adults, healthy elders and mild cognitive impairment patients. Brain Imaging Behav. 12, 127–141. 10.1007/s11682-017-9686-y 28176262

[B7] Esposito R. Cieri F. di Giannantonio M. Tartaro A. (2018). The role of body image and self-perception in anorexia nervosa: the neuroimaging perspective. J. Neuropsychol. 12, 41–52. 10.1111/jnp.12106 27220759

[B8] Raichle M. E. MacLeod A. M. Snyder A. Z. Powers W. J. Gusnard D. A. Shulman G. L. (2001). A default mode of brain function. Proc. Natl. Acad. Sci. U.S.A. 98, 676–682. 10.1073/pnas.98.2.676 11209064 PMC14647

[B9] Welch E. Jangmo A. Thornton L. M. Norring C. von Hausswolff-Juhlin Y. Herman B. K.. (2016). Treatment-seeking patients with binge-eating disorder in the Swedish national registers: clinical course and psychiatric comorbidity. BMC Psychiatry 16:163. 10.1186/s12888-016-0840-7 27230675 PMC4880842

